# Attempted Suicide by *Cerbera odollam* Ingestion in Switzerland: A Case Report

**DOI:** 10.1155/crcc/3846219

**Published:** 2025-09-03

**Authors:** Valerie Girsberger, Sandra Koller-Palenzona, Alexander Jetter, Marie-Elisabeth Kajdi

**Affiliations:** ^1^ Intensive Care Unit, Kantonsspital Graubunden, Chur, Switzerland; ^2^ Tox Info Suisse, Swiss National Poison Center and Associated Institute of the University of Zurich, Zurich, Switzerland

## Abstract

We report the case of a 37‐year‐old female patient admitted to our hospital with unstable bradyarrhythmia, abdominal cramping, vomiting and visual disturbances including xanthopsia and diplopia. Ten hours prior to admission, she reported ingestion of four seeds of *Cerbera odollam* and four crushed nutmegs with suicidal intent. The patient was hypotensive and suffered from hyperkalemia (serum potassium > 5.5 mmol/L), leucocytosis and acute kidney injury. Her heart rate ranged between 28 and 40 bpm, and electrocardiogram (ECG) revealed a second‐degree atrioventricular (AV) block and ST‐depressions. The patient developed a third‐degree AV block requiring vasopressors and transvenous pacing, which she remained completely dependent upon for 56 h. After a total of 3 days, the patient remained hemodynamically stable, and the pacemaker was removed. ECG still featured downsloping ST‐depressions, first‐degree AV block and sinus bradycardia. The patient survived. Her clinical course was typical and severe. *Cerbera odollam* poisoning is a potentially fatal condition associated with arrhythmia, abdominal symptoms and electrolyte disturbances. It remains a rarity in Europe and hardly known to Western physicians. Given its potentially lethal effects and increasing availability of its seeds, *Cerbera odollam* poisoning must be included in the differential diagnosis of unknown intoxications. With this case report, we hope to raise awareness among physicians for *Cerbera odollam* intoxications in Europe.

## 1. Background

*Cerbera odollam* is a tree indigenous to Southeast Asia, Madagascar and India. It is also commonly referred to by alternative names such as “pong‐pong” or “suicide‐tree” [1]. It yields fruits with a highly furrowed endocarp‐covered seed of up to 10 cm in diameter. The whole plant is toxic, particularly the seeds. The primary toxic agent is cerberin, a cardiac glycoside with a mechanism of action similar to digoxin. It causes disruption of cardiac electrical conduction and hyperkalemia by binding to and inhibiting sodium–potassium adenosine triphosphate (Na+/K+ ATPase) exchanger in myocardial cells [1]. Ingestion typically results in gastrointestinal symptoms such as nausea, vomiting and diarrhea, as well as various degrees of bradycardia, heart block and hyperkalemia [2–7].

According to Gaillard et al., no other plant is associated with as many cases of intentional poisoning. In the Indian state of Kerala, the seeds are responsible for 50% of plant poisonings, causing an average of 50 deaths a year. Notably, approximately 70% of the victims are women [3].

Compared to its countries of origin, the plant and its use for homicidal and suicidal attempts are hardly known in Western countries. Nonetheless, the existing literature describes an increasing number of cases in the United States over the past decade [2, 4, 8–11]. Data on intoxications occurring in Europe are scarce, and only a single case was described in Austria [12].

To our knowledge, this is the first documented case of intoxication with *Cerbera odollam* occurring in Switzerland. Our patient imported the poisonous seeds directly from a holiday in India. The internet as another source for purchasing seeds has been described in the literature [2, 4].

The objective of this report is to increase awareness among clinicians regarding the clinical presentation and appropriate management of *Cerbera odollam* toxicity.

## 2. Case Presentation

A 37‐year‐old female patient was admitted to the emergency department of a tertiary care hospital by ambulance presenting with unstable bradyarrhythmia. She also suffered from central neurologic symptoms with visual disturbances including xanthopsia, diplopia, flickering, blurred vision and dizziness, as well as abdominal symptoms including nausea with hypersalivation, excessive, repetitive vomiting and abdominal cramping. Approximately 10 h prior to admission, the patient had ingested *Cerbera odollam* seeds and crushed nutmegs in what was her first suicide attempt. Past medical history included chronic pain secondary to cervical disc herniation with cervical myelopathy and depressive adjustment disorder. Approximately 6–8 h after ingestion, her symptoms progressively worsened, prompting her to contact emergency medical services.

On physical examination upon arrival, the patient was agitated, dizzy, and uncooperative, yet awake and oriented to person, place, and time with a Glasgow Coma Scale of 15. The patient’s pupils were equally round and reactive to light. Cardiovascular examination revealed bradycardia, absence of murmurs and capillary refill time of less than 3 s. The skin was dry and cool on the extremities. Abdominal examination revealed mild diffuse tenderness and the presence of bowel sounds. The remainder of the physical examination was noted as within normal limits.

**Figure 1:**
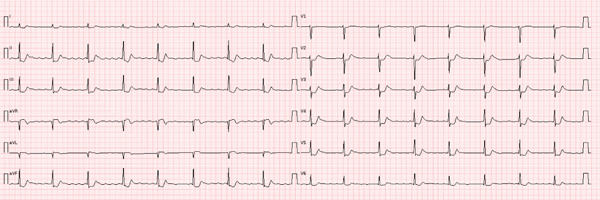
Electrocardiogram (ECG) on admission with second‐degree atrioventricular block and ST‐depressions (velocity 25 mm/s).

Vital signs revealed bradycardia with heart rates ranging from 28 to 40 bpm, body temperature of 36.8°C and respiratory rate of 18 breaths per minute with pulse oximetry of 98% on room air. After a brief hypertensive episode with a peak blood pressure of 181/117 mmHg, she developed hypotension. The initial electrocardiogram (ECG) revealed a second‐degree atrioventricular (AV) block (Mobitz Type II) with alternating conduction ranging between 3:1 and 5:1 resulting in bradycardia around 30 bpm (Figure 1). Laboratory findings included leucocytosis (25.6*e* ^3^/*μ*L), hyperkalemia (6 mmol/L), and acute renal insufficiency classified as acute kidney injury network (AKIN) Stage I with a serum creatinine of 119 *μ*mol/L and a baseline creatinine of 60 *μ*mol/L. A digoxin immunoassay from the patient’s serum revealed a digoxin serum concentration of 0.89 nmol (therapeutic range 1–2.6 nmol/L).

According to the patient, a total of four “small” seeds had been ingested along with four previously crushed nutmegs. Specifications regarding the origin of the seeds given by the patient varied. After initially claiming to have purchased them online, she later referred to a recent trip to India, during which she had collected the seeds with suicidal intent.

### 2.1. Differential Diagnosis

The abdominal symptoms, including nausea and severe vomiting, can be attributed to intoxication with either nutmeg or *Cerbera odollam*. The visual disturbances, as well as agitation are more commonly associated with nutmeg toxicity. Bradycardia and high‐degree AV block, as well as ST‐depressions, are characteristic features of *Cerbera odollam* poisoning [1, 3, 13–17].

### 2.2. Treatment and Clinical Course

Initial treatment in the emergency department included the administration of intravenous atropine (0.5 mg) and ephedrine (with a cumulative dose of 30 mg). After consultation with the national center for the treatment of intoxications (Tox Info Suisse), 40 mg of digoxin‐specific antibody fragments (digoxin immune fab, DigiFab, BTG Pharmaceuticals, West Conshohocken, PA, United States)—an antidote primarily developed for the treatment of digoxin intoxication—was administered. Hyperkalemia was managed by promoting the intracellular shift of potassium through insulin–glucose infusion. Afterward, the patient was transferred to the intensive care unit (ICU).

**Figure 2:**
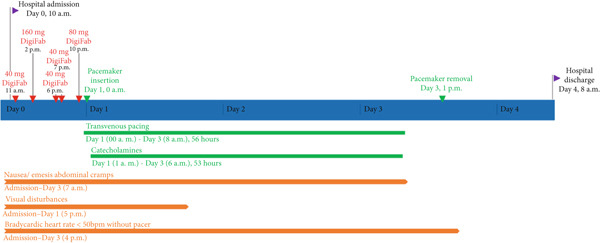
Timeline after ingestion of *C. odollam* and nutmeg seeds. DigiFab, digoxin immune fab.

Two hours after admission, the patient’s hemodynamic status deteriorated with progression to third‐degree AV block. The effect of atropine was insufficient and short‐lasting, necessitating a cumulative dose of 6 mg within 6 h. Subsequently, a total of 320 mg digoxin‐specific antibody fragments were administered over the next 8 h (for details, please refer to Figure 2) without significant effect on heart rate, which remained between 35 and 50 bpm. In the absence of clinical improvement, a decision was made to insert a transvenous cardiac pacemaker 24 h after ingestion. The pacing rate was set to 70 bpm and after insertion, the patient entirely depended on the pacemaker for 56 h. During this time period, the patient additionally required continuous application of norepinephrine in order to treat persistent hypotension. No further antibodies were administered after pacemaker insertion. Nausea was treated successfully with dexamethasone and metoclopramide.

**Figure 3:**
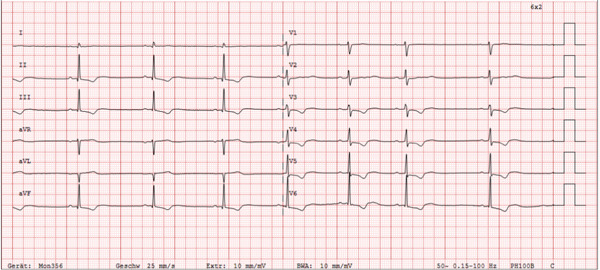
ECG on Day 3 with bradycardic sinus rhythm and characteristic downsloping ST‐depression (velocity 25 mm/s).

After 3 days of supportive care, catecholamines were discontinued, and the pacemaker was temporarily paused. The patient exhibited a bradycardic sinus rhythm with a heart rate of 48 bpm with presence of first‐degree AV block. The patient remained hemodynamically stable with normotensive blood pressure, allowing for uncomplicated removal of the transvenous pacemaker. The ECG featured characteristic downsloping ST‐segment depression (Figure 3).

### 2.3. Outcome and Follow‐Up

Following initial stabilization, the patient remained normotensive, alert and cooperative and was finally transferred to the psychiatric ward on Day 4 of hospitalization due to the persistent risk of self‐harm. The patient was readmitted to our hospital only shortly after within 24 h after discharge from the ICU due to the recurrence of the third‐degree AV block. She denied new ingestion of *Cerbera odollam* seeds. As she remained hemodynamically stable with normotensive blood pressure and symptom‐free, she could be transferred to the general ward under continuous telemetric monitoring and did not require further treatment in the ICU. The AV block resolved completely within 3 days.

## 3. Discussion

To our knowledge, this is the first reported case of *Cerbera odollam* intoxication in Switzerland. Currently, *Cerbera odollam* poisoning is rare in Europe and therefore relatively unknown to Western physicians [4, 12, 18, 19]. However, due to globalization—particularly in the context of international travel and the accessibility of toxic substances via the internet—the incidence of intoxication is likely to increase. Regarding the literature, clinical data on *Cerbera odollam* intoxication are limited to case reports and a small number of retrospective studies. Our patient presented with nausea, vomiting, abdominal cramping, symptomatic bradycardia, high‐degree AV block and hyperkalemia. This constellation of symptoms is consistent with a typical and severe clinical course [1, 3, 20]. Renymol et al. described abdominal pain, diarrhea, vomiting, drowsiness, bradycardia and hypotension as well as ECG changes as typical symptoms after ingestion. In their retrospective analysis, a total of 102 patients were included, of whom 24 presented with hyperkalemia > 5.5 mmol/L [19].

In our case, the patient was able to disclose both the type and quantity of seeds ingested upon admission. At present, there is no test available to formally identify cerberin—the active compound of *Cerbera odollam*—for diagnostic or forensic purposes [1, 3, 21]. Since it shares structural similarities with digoxin, including a four‐carbon ring steroid‐type structure with a lactone ring and an added sugar remnant, immunoassays designed to detect the steroid‐type structure of digoxin will likely react in the presence of cerberin. Nevertheless, these assays do not allow for specific identification of the molecule or quantification [1, 3]. More advanced techniques, such as liquid chromatography–tandem mass spectrometry (LC–MS/MS), may be employed for cerberin detection in blood, although such methods are not routinely available.

Based on the literature, we assumed that the patient might not have survived the incident in a setting of poor resources, particularly without the option of temporary cardiac pacing. Ingestion of more than two kernels, late presentation to hospital—defined as time from ingestion to hospital admission of greater than 6 h—presence of nausea, bradycardia, hypotension, and severe ECG changes are associated with significantly higher mortality [19]. Administration of digoxin‐specific antibody fragments, an expensive and controversial treatment of *Cerbera odollam* intoxication [2, 8, 9, 22, 23], did not result in clinical improvement in our patient.

Recurrence of third‐degree AV block on Day 4 seems likely to be associated with initial *Cerbera odollam* ingestion. Retrospectively, the transfer to the psychiatric ward might have been too early, as the patient continued to exhibit bradycardic sinus rhythm and downsloping ST‐depressions.

## 4. Conclusion

*Cerbera odollam* poisoning is a potentially fatal condition characterized by arrhythmia, gastrointestinal symptoms, and electrolyte disturbances. Given its potentially lethal effects and increasing availability in Europe, *Cerbera odollam* poisoning should be considered in the differential diagnosis of unknown intoxications presenting with any of these clinical features.

Knowledge about clinical presentations, complications, and treatment options is crucial for the patient’s survival. Triage must consider local healthcare infrastructure, and optimal treatment necessitates transfer to a facility equipped with an ICU, availability of transvenous cardiac pacing, and renal replacement therapy. With this case report, we aim to raise awareness among physicians and forensic toxicologists for the increasing incidence of cerberin intoxications in Europe.

## Consent

Written informed consent for publication was obtained from our patient.

## Conflicts of Interest

The authors declare no conflicts of interest.

## Funding

No funding was received for this manuscript.

## Data Availability

All relevant data supporting the findings of this case report are included within the article.
